# Comparative assessment of regional tau distribution by Tau-PET and Post-mortem neuropathology in a representative set of Alzheimer’s & frontotemporal lobar degeneration patients

**DOI:** 10.1371/journal.pone.0284182

**Published:** 2023-05-11

**Authors:** Rodolfo G. Gatto, Arenn F. Carlos, R. Ross Reichard, Val J. Lowe, Jennifer L. Whitwell, Keith A. Josephs

**Affiliations:** 1 Department of Neurology, Mayo Clinic, Rochester, MN, United States of America; 2 Department of Laboratory Medicine and Pathology, Mayo Clinic, Rochester, MN, United States of America; 3 Department of Radiology, Mayo Clinic, Rochester, MN, United States of America; National Center of Neurology and Psychiatry (NCNP), JAPAN

## Abstract

Flortaucipir (FTP) PET is a key imaging technique to evaluate tau burden indirectly. However, it appears to have greater utility for 3R+4R tau found in Alzheimer’s disease (AD), compared to other non-AD tauopathies. The purpose of this study is to determine how flortaucipir uptake links to neuropathologically determined tau burden in AD and non-AD tauopathies. We identified nine individuals who had undergone antemortem tau-PET and postmortem neuropathological analyses. The cohort included three patients with low, moderate, and high AD neuropathologic changes (ADNC), five patients with a non-AD tauopathy (one Pick’s disease, three progressive supranuclear palsies, and one globular glial tauopathy), and one control without ADNC. We compared regional flortaucipir PET uptake with tau burden using an anti-AT8 antibody. There was a very good correlation between flortaucipir uptake and tau burden in those with ADNC although, in one ADNC patient, flortaucipir uptake and tau burden did not match due to the presence of argyrophilic grains disease. Non-AD patients showed lower flortaucipir uptake globally compared to ADNC patients. In the non-AD patients, some regional associations between flortaucipir uptake and histopathological tau burden were observed. Flortaucipir uptake is strongly linked to underlying tau burden in patients with ADNC but there are instances where they do not match. On-the-other hand, flortaucipir has a limited capacity to represent histopathological tau burden in non-AD patients although there are instances where regional uptake correlates with regional tau burden. There is a definite need for the development of future generations of tau-PET ligands that can detect non-AD tau.

## Introduction

The misfolding and alternative splicing of tau proteins are key molecular features of Alzheimer’s disease (AD) and other related tauopathies [1]. The development of tau-specific positron emission tomography (PET) tracers, which are now available for clinical evaluation, has been a significant breakthrough in AD research and imaging patient assessment [2–4]. In 2020, the U.S. Food and Drug Administration approved the use of a tau ligand (Flortaucipir) as a ligand to detect a phosphorylated tau fraction (epitope known as phosphorylated para-helicoidal filament one or PHF-1) [5]. Some studies using tau PET have achieved good diagnostic differentiation between AD and other degenerative diseases such as frontotemporal lobar degeneration (FTLD) [6,7]. Conversely, ex-vivo studies have shown that flortaucipir lacks sensitivity to detect pathology in FTLD with a large inter- and intra-patient variability [8]. Moreover, whereas some studies showed a good correlation between flortaucipir uptake and measurements of hyperphosphorylated 4-repeat (4R) tau immunostained across regional tissues [9,10], others performing semiquantitative regional lesion counts in autopsied 4R tauopathies did not support flortaucipir having affinity to 4R tau [11]. In addition, autoradiography flortaucipir studies have found little-to-no binding of flortaucipir to 4R tau or 3R tau which have generated questions related to the affinity of flortaucipir for such tau lesions [12,13]. To addresses some of these discrepancies between tau PET and underlying tau pathology we aimed to assess the relationship between flortaucipir uptake and tau burden in the cases of AD and 3R+4R pair helical filament tau versus those with non-AD 3R or 4R tauopathies.

Since the implementation of the Braak neurofibrillary tangle (NFT) staging system to show the distribution of tau deposition in AD [14], neuropathological evaluation has relied on semiquantitative scoring systems measuring the number of tau inclusions observed in neuronal and/or glial cells [15]. Such methods, however, do not account for the aggregated role of tau burden nor the functional importance of tau deposition across each area of the brain [16]. Still, the variability of first-generation tau PET ligand binding in dementia has been histologically studied with *ex vivo* radiolabeling trying to mirror the *in vivo* pattern of the flortaucipir tracer [8]. Interestingly, microscopic analysis of the pathological phospho-labeled tau inclusions revealed that these tracers preferentially bind to premature tau aggregates [8]. In these autoradiographic studies, molecular binding only labeled neuronal tau in symptomatic disease without accounting for regional factors such as blood flow or neuroinflammation. Furthermore, none of these ligands were able to determine the presence of these analogs in glial cells, which are particularly important in non-AD tauopathies with high heterogeneity in cellular tau expression [17]. This lack of correlation between pathological tau burden and tracer binding supports rigorously assessing current and novel tau PET tracers before translating them into clinical studies, especially in non-AD tauopathies [18]. More recently, a growing body of work has been published suggesting the detection of tau in non-AD, with ligands such as 18F-PI-2620 [19] and 18F-PM-PBB3 [20]. Some studies have also examined ligand detection in patients with progressive supranuclear palsy (PSP), a type of motor FTLD, using PM-PBB3 [21] or 18F-PI-2620 [22].

Neuropathology remains the current gold standard to assess tau content in brain tissue with a high level of structural information [23]. However, in patients with a lower expression of microscopic tau, neuropathology may not allow for the full appreciation of its distribution. In such patients, it may be necessary to increase the number of sampled gray- and white-matter regions to improve accuracy [24]. A larger number of neuronal markers and brain samples implies a need for automatic imaging techniques to expedite classification, segmentation, and data extraction techniques [25]. This has resulted in new machine-learning approaches using novel markers [26].

This study aims to determine the relationship between flortaucipir PET uptake and tau burden assessed quantitatively by postmortem neuropathological studies in patients with varying degrees of AD neuropathological changes (ADNC), and non-AD 3R and 4R tauopathies. We hypothesize that flortaucipir PET uptake will correlate better with tau burden in cases with ADNC compared to those with non-AD tau.

## Materials and methods

### Patient selection

We queried the Mayo Clinic neuropathological database in Rochester, Minnesota, for all patients who had been enrolled into an NIH-funded study by the Neurodegenerative Research Group (NRG; PIs: Josephs & Whitwell); had died with brain autopsy examination between January 1, 2017, and July 31, 2022; and had completed an antemortem flortaucipir PET scan. We identified a total of nine patients. Of these, three were pathologically diagnosed as having AD neuropathological changes (ADNC): One had evidence of low likelihood ADNC (Low-AD); one had intermediate likelihood ADNC (Int-AD), and the third had a high likelihood of ADNC (High-AD) [27]. Five patients had a non-AD tauopathy, including three with progressive supranuclear palsy (PSP) with an antemortem clinical diagnosis of PSP-Richardson syndrome (PSP-RS) in one and PSP with predominant speech/language impairment (PSP-SL) in two, and one each with Pick’s disease (PiD) and glial globular tauopathy (GGT). The ninth patient had neither ADNC, non-AD tau, nor a degenerative disease but was diagnosed with mitochondrial encephalopathy lactic acidosis, and strokes (MELAS). Hence, this patient was used as a control.

The study was approved by the Mayo Clinic IRB, and all patients or proxies consented to the research study. (The proxies provided consent for patients when needed.) The study followed the ethical standards of the Committee on Human Experimentation at Mayo Clinic by the Helsinki Declaration of 1975.

### Neuropathological evaluation

All patients had a standardized neuropathologic evaluation following accepted, published methodologies [27,28]. Demographic details, including the degree of ADNC in each patient, can be seen in **Table 1.** Specifically, our study included a tau antibody (Invitrogen, MN#1020; Phospho-Tau (Ser202, Thr205) AT8 clone; mouse monoclonal at 1:250) to stain key brain regions affected in tauopathies. From previous studies [29], a substantial portion of the AT8 epitope is phosphorylated derived from PHF-1 in immature brains [30,31], but only a modest fraction of AT8 resides in the adult normal brain [32]. For each patient, we included brain block sections from neuropathological established ROIs, including the amygdala, hippocampus, lateral middle temporal lobe, cingulum, superior frontal lobe, inferior parietal lobe, occipital lobe, basal ganglia, thalamus, and midbrain, according to standard neuropathologic examination following cortical sampling according to the Consortium to Establish a Registry for Alzheimer’s disease (CERAD) [33]. To assess molecular changes in tau burden, we applied DAB (3,3’-diaminobenzidine) staining with a mouse-host monoclonal Phospho-PHF-tau pSer202+Thr205 antibody, clone AT8, (Thermo Fisher Scientific, MN1020, 1:100). We used standard procedures to establish tau-burden scores on each selected brain sample for each patient. We performed quantitative histological analysis using threshold and masking methods with ImageJ [34,35]. The percentage of ROI areas from all ROIs were thresholded, masked, globally averaged, and plotted against the values on each ROI. Additional semiquantitative scores to assess tau burden were based on four types of lesions (neurofibrillary tangles, coiled bodies, astrocytic lesions, and neuropil threads) cataloged on a 10x magnification field (200x total). Imaging was performed by a regular scanning microscope (Grundium Ocus®40 Digital pathology microscope scanner). Four different, randomly selected sub-regions in each histology section were visually cataloged and averaged to obtain a final score per ROI described elsewhere [36]. A final global score across all ROIs was further calculated for each patient.

**Table 1:** Demographic and clinical features.

Patient	Neuropathological diagnosis *	Sex	Age onset	Age at tau PET scan	Age at death	Braak Stage	Thal phase	ADNC
**1**	MELAS (non-Tau Control)	F	69	70	71	0	0	A0 B0 C0
**3**	ADNC (Low-AD)	M	64	76	80	I- II	I-II	A1 B1 C0
**2**	ADNC (Int.- AD)	F	69	74	79	III	III	A2 B2 C3
**4**	ADNC (High-AD)	M	58	75	78	VI	IV	A3 B3 C3
**5**	Typical PSP (PSP- RS)	M	64	66	68	I	0	A0 B1 C0
**6**	Atypical PSP (PSP-SL)	M	73	82	82	III- IV	III	A2 B2 C3
**7**	Atypical PSP (PSP-SL)	M	72	82	83	II	0	A0 B1 C0
**8**	Pick’s body disease	F	63	71	75	0	0	A0 B0 C0
**9**	Globular glial tauopathy- type I	F	81	83	87	II	III	A2 B1 C1

### Tau PET neuroimaging

All flortaucipir scans were acquired using a GE PET/CT scanner (GE Healthcare, Milwaukee, Wisconsin). Patients were injected with ~370MBq (range 333–407MBq) of [18F] flortaucipir, followed by a 20-minute PET acquisition performed 80 minutes after injection; 20-minute late-uptake PET scans consisted of four 5-minute dynamic frames. PET sinograms were reconstructed with OEM into a 256mm FOV (pixel size = 1.0mm, slice thickness = 3.3mm). The four individual frames were averaged for analysis. All patients underwent a 3T MRI protocol on a GE scanner that included magnetization-prepared, rapid gradient-echo (MPRAGE) (TR/TE/T1 = 2300/3/900ms; 26-cm FOV, slice thickness = 1.2mm, in-plane resolution = 1mm) and fluid-attenuated inversion recovery (FLAIR) (TR/TE = 11000/147ms; 22-cm FOV; slice thickness = 3.6mm) sequences. Whitwell and colleagues previously described further details of the methods and procedures [18]. We calculated regional flortaucipir uptake for a set of regions of interest (ROIs) matching the neuropathological regions. Normative parameters propagated to the patient’s individual MPRAGE space were used to create standardized uptake value ratios (SUVRs) with regional values on DSI Studio (https://dsi-studio.labsolver.org/). Median flortaucipir uptake was calculated across gray and white matter in each ROI, and median values were normalized by median uptake in cerebellar crus gray matter. Each patient’s output images were co-registered again on DSI Studio [37] using a rigid-body registration type with 200 random search iterations and a mutual iteration cost function. This coregistration established a new set of optimized translocations, scaling, shearing, and rotation coordinates to adjust the coregistration. Detailed descriptions of the initial signal extractions and PET signal processing have been previously published [38,39]. A corrected SUVR exceeding 1.25 on each ROI was considered abnormal (vertical red line displayed in **Figs 2 & 5**) following previously established guidelines [40]. We supervised these spatial modifications interactively by visual inspection to avoid misalignment due to extreme neuroanatomical distortions. At this point, we reacquired ROI parcellations from the AAL2 atlas [41]. We added regional ROIs to create merged lobar regions, including the left and right sides of the brain, to match neuropathological ROIs. Regions selected to match ROIs were assessed pathologically. Averaged left- and right-sided segmented ROIs included the amygdala, hippocampus, temporal lobe, cingulum, frontal lobe, parietal lobe, occipital lobe, basal ganglia, and thalamus. Midbrain and pons regions were segmented using the FreeSurfer atlas. However, we excluded these regions from our correlative analysis. For correlative analysis, right (R) and left (L) ROIs from the Tau PET were specifically correlated to the side of the neuropathological ROI.

### Statistical analysis

Comparative analysis from each region-specific tau PET SUVRs from the AD and non-AD groups were matched with their corresponding percentage of AT8 areas calculated as previously described in the quantitative histopathological method section. Non-parametric Spearman rank correlation coefficients (rho), between the percentage of AT8 area on histopathology and TauPET imaging (SUVRs), were calculated for AD and non-AD groups.

## Results

### Patient population

At the time of flortaucipir PET scans, there were no age differences between the ADNC group (73.8 +/- 3.6 y.o.) and the non-AD group (75.5 +/- 8.3 y.o.), (P>0.88; **Table 1).** Of the three patients with PSP pathology, one with PSP-RS showed typical PSP pathology while the other two with PSP-SL showed atypical PSP pathology with more cortical 4R tau deposition.

### Tau PET measurement

**Fig 1:**
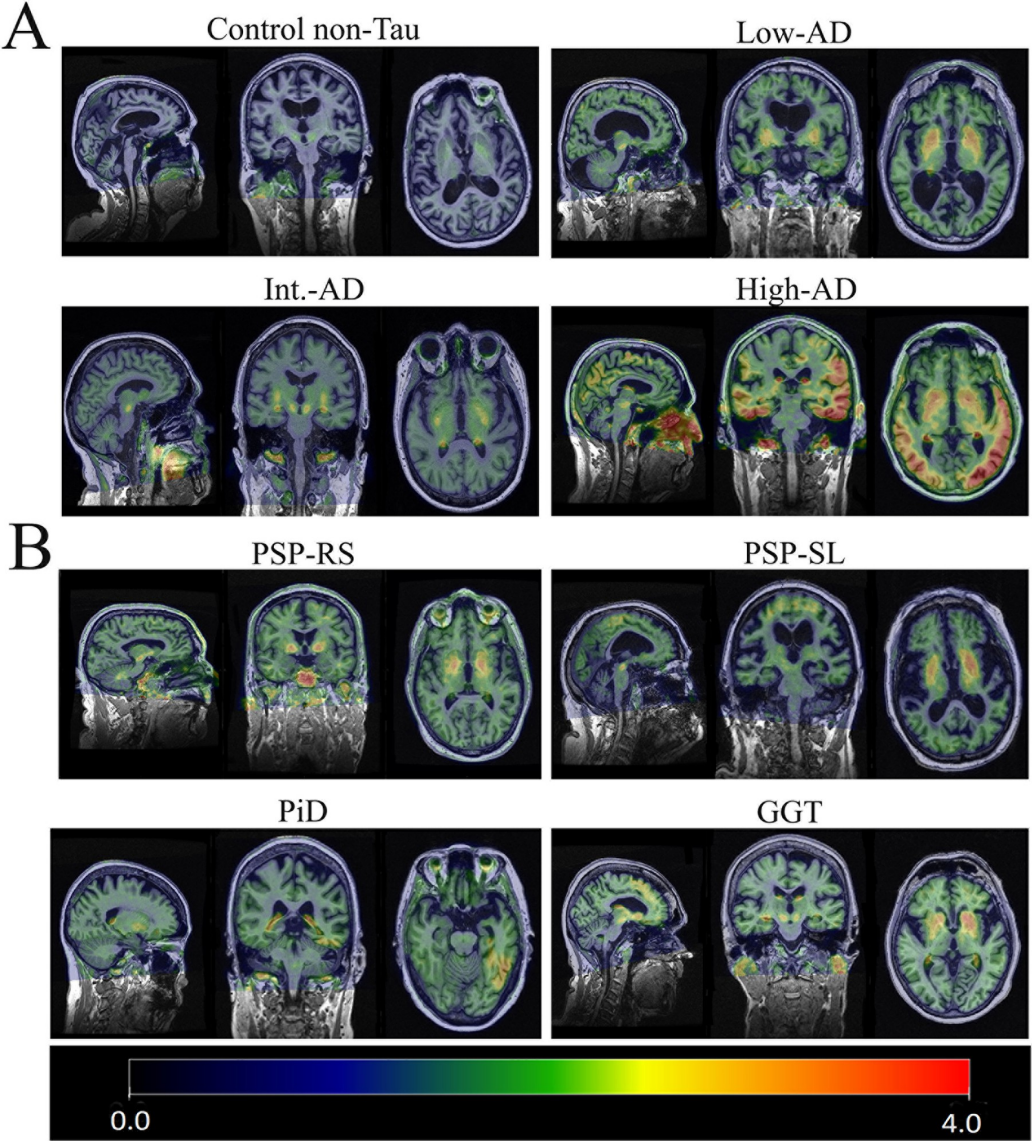
Tau PET studies from the representative patients included in this study. **A-** Tau PET images from non-tau control and patients with Alzheimer’s disease (AD) and global low, moderate, and high degrees of tau PET ligand. B- Tau PET images from non-AD patients.

**Fig 2:**
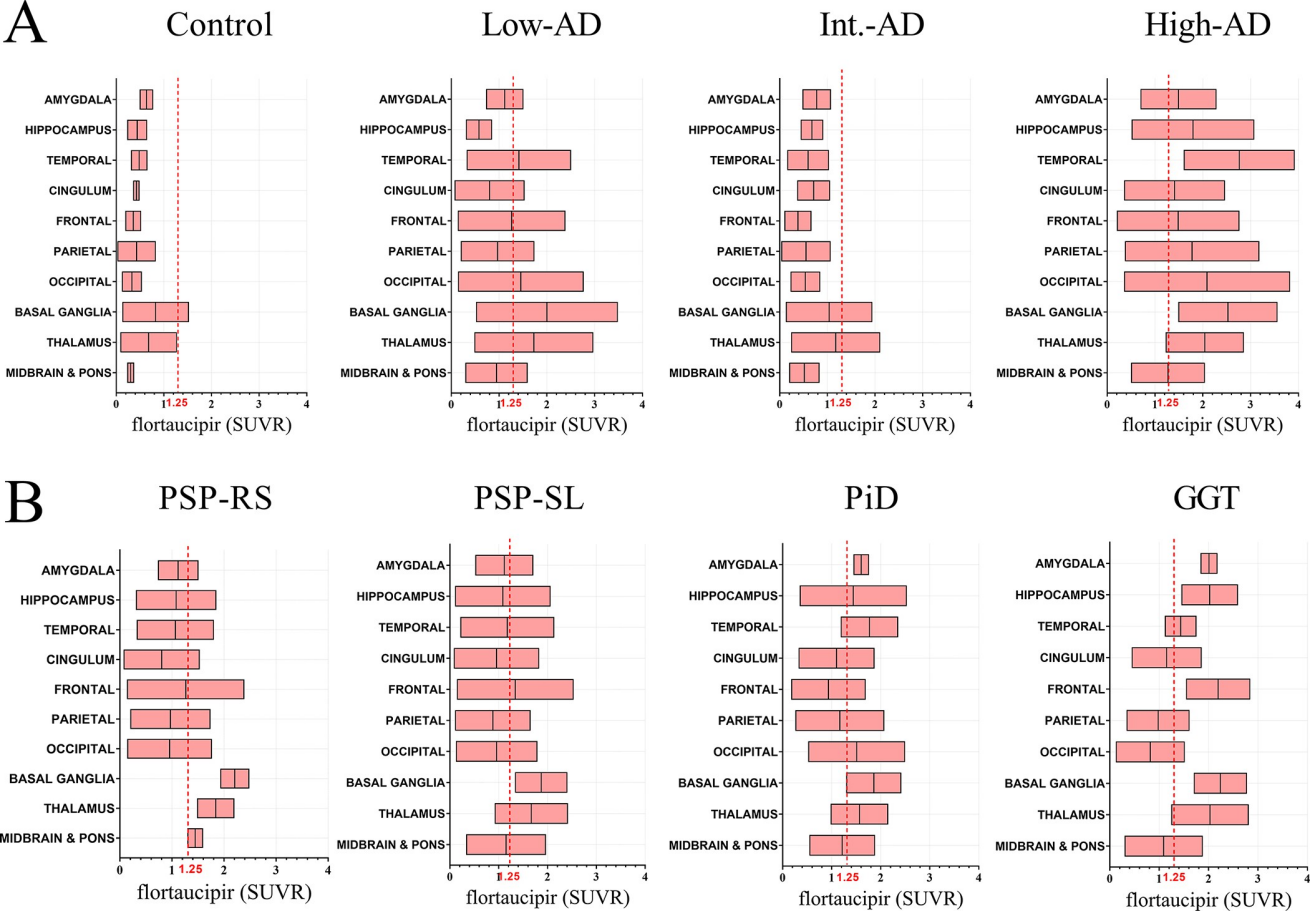
Tau PET Standardized Uptake values (SUVRs) in selected ROIs matching neuropathological studies. The red vertical line (SUVR = 1.25) represents the Tau PET detected concentration as established normal levels. **Abbreviations:** AD, Alzheimer’s Disease; PSP-RS, progressive supranuclear palsy—Richardson’s syndrome; PSP-SL, progressive supranuclear palsy speech/language variant; PiD, Pick’s disease; GGT, glial globular tauopathy.

**Fig 1** displays flortaucipir uptake in all patients. No cortical flortaucipir uptake was observed in the control patient or the low-AD patient. Elevated cortical flortaucipir uptake was observed in the intermediate-AD and high-AD patients, particularly involving the temporal lobe. Areas of increased uptake were observed in the basal ganglia of all nine patients, including the non-tau control patient, likely as a result of the non-specific binding of the flortaucipir in deep gray matter (GM) regions. Flortaucipir uptake in the non-AD tauopathies was generally lower than in the intermediate-AD and high-AD patients. Overall, patterns of flortaucipir uptake were heterogeneous across the non-AD tauopathies with different distributions for each pathology **(Fig 2)**.

### Quantitative and semi-quantitative tau burden measurements by immuno-histochemistry

**Fig 3:**
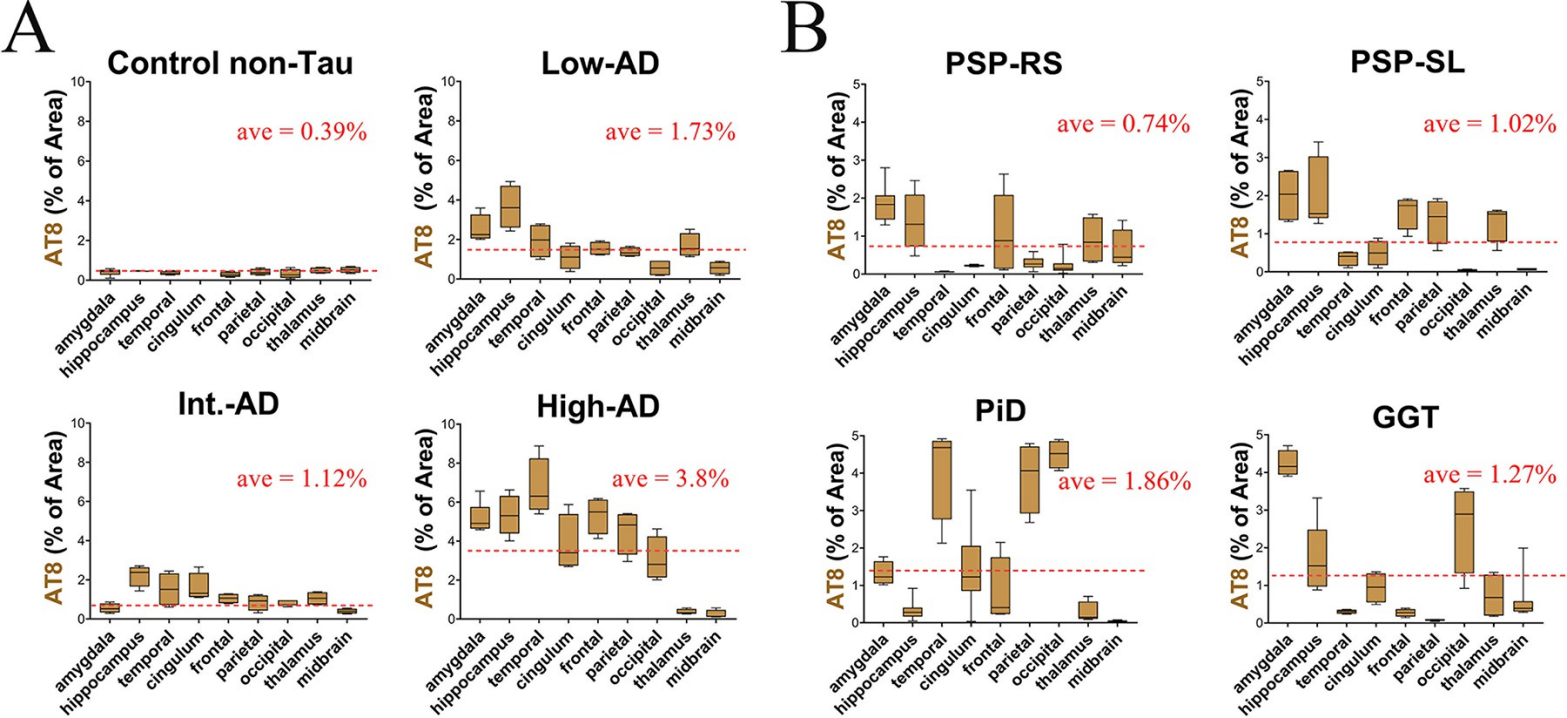
Quantitative evaluation of histological tau burden in brain samples in AD and non-AD groups. **A—**Quantitative Evaluation of immature histopathological tau (clone AT8) in control and AD group. Note a progressive intraindividual increase in AT8 (above the red dotted line) **B—**Quantitative analysis of the non-AD group showing a more heterogeneous increase in AT8 across different brain regions. Representative pictures from comparative frontal, temporal, and occipital regions, show the neuropathological variability of AT8 distribution.

A quantitative-scoring system showed that global tau burden increased from the control patient to the low-AD, intermediate-AD, and high-AD Tau PET uptake patients **(Fig 3)**. The non-AD tauopathies showed a lower global tau burden than the AD-high patient. Among the three AD-spectrum patients, the high-AD patient had the highest global tau burden, particularly in the temporal lobe and hippocampus. The low-AD and intermediate-AD patients also showed the greatest regional tau burden in the temporal lobe and hippocampus, although with a lower AT8 burden than observed in the high-AD patient. Across the non-AD tauopathies, patterns of regional tau burden were inconsistent by region and heterogeneous. The three PSP patients showed increased AT8 tau burden in the medial temporal lobe, frontal lobe, and thalamus. The PiD patient showed the highest AT8 burden in cortical regions, whereas the GGT patient showed a high AT8 burden in the amygdala.

**Fig 4:**
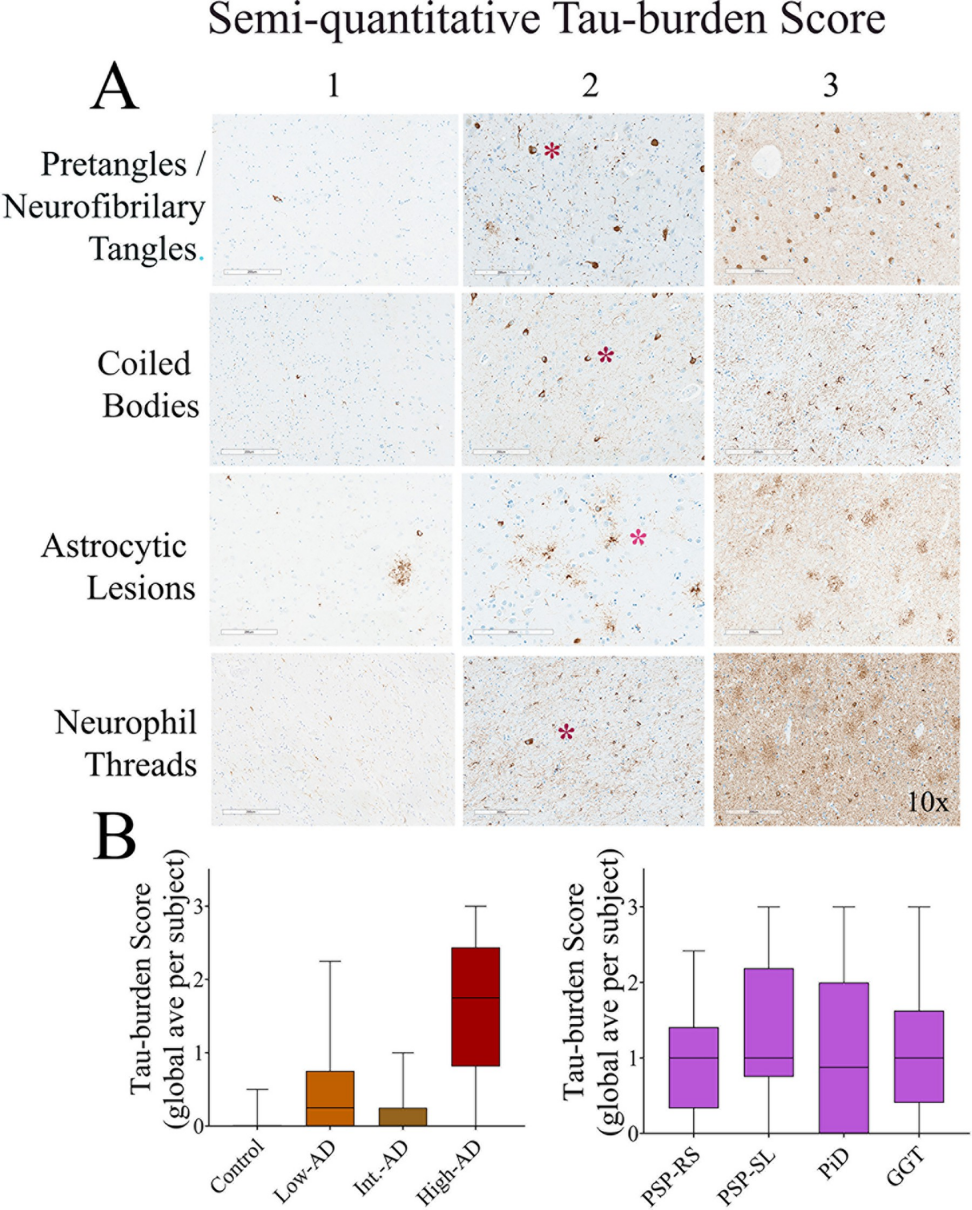
Semi-quantitative neuropathological scoring evaluation of tau burden. **A**—Summary of a categorical scoring system used to calculate tau burden based on four subscores: **1) Pre-tangles & neurofibrillary tangles** (0, none; 1, one to three; 2. three to seven; 3, more than seven). **2) Coiled bodies** (0, none; 1, one to three; 2. three to seven; 3, more than seven). **3) Astrocytic lesion** (0, none; 1, one to three; 2. three to seven; 3, more than seven). **4) Neurophil threads** (0, none; 1, mild density; 2, moderate density; 3, high density). **B—**Global averaged tau scores across all ROIs.

**Fig 5:**
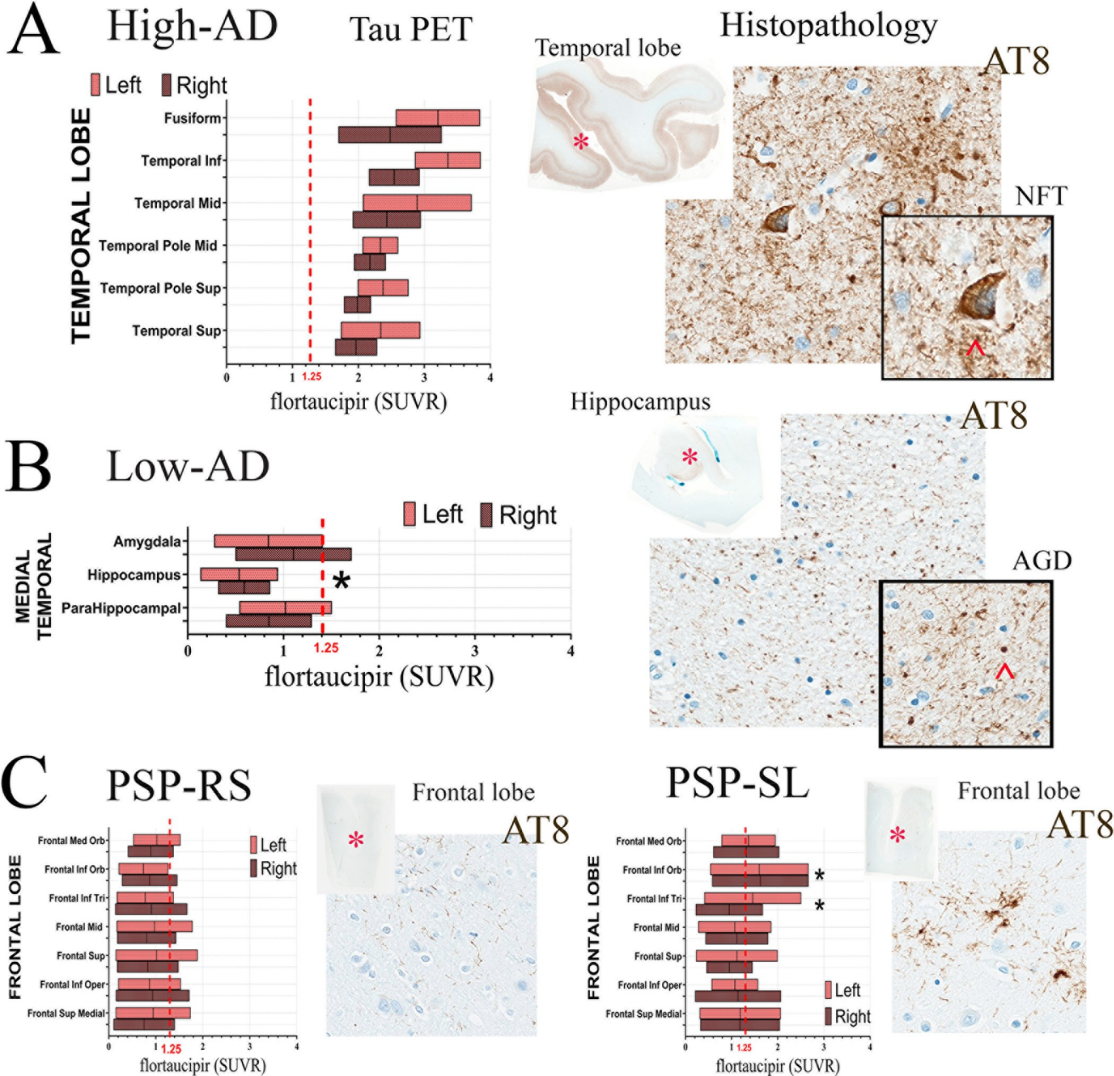
Comparison between tau PET signal and histopathological findings in AD and non-AD tauopathy patients. **A** -tau PET plots from our AD patient with overall low tau show significantly lower SUVR levels in the left and right hippocampus compared to other medial temporal regions. However, the postmortem histopathological examination shows the presence of abundant intracellular immature tau (AT8) as an example of the mismatch between tau PET and histopathology in low flortaucipir-TAU regimes. **B—**A plot from an AD patient with high tau demonstrates a good match between tau PET and AT8 histopathology in cortical areas of the temporal lobe (right). However, in non-AD tauopathies such as PSP-RS and PSP-SL, there is a mismatch between tau PET and neuropathological findings. This could imply the association of the ligand to cellular types not necessarily linked to tau aggregation (off-target binding). The scale bar length represents 200 microns.

The semi-quantitative evaluation of different types of tau lesions is shown in **Figs 4 and 5**. Whereas the overall compiled scores in the four non-AD patients demonstrated elevated tau burden scores **(Fig 4**), only the high-AD patient showed a definitive elevated tau pattern compared to the control patient. The low-AD and moderate-AD patients showed minimally elevated tau compared to the control patient.

### Comparisons between flortaucipir PET and histopathological tau burden

In the AD-high patient, there was good correspondence between flortaucipir uptake and histopathological tau burden (**Fig 5**). However, in the low-AD patient, a mismatch was observed between flortaucipir PET and histopathological tau burden whereby the hippocampus showed immature intracellular tau (AT8) on pathological examination but did not show elevated flortaucipir uptake on PET (**Fig 5**). PSP-SL patients had slightly higher frontal flortaucipir levels than PSP-RS patients. This fits nicely with pathological findings of slightly more tau on AT8 in the PSP-SL patients compared to the PSP-RS patient, in the frontal lobe.

### Flortaucipir PET and histopathology correlations

**Fig 6:**
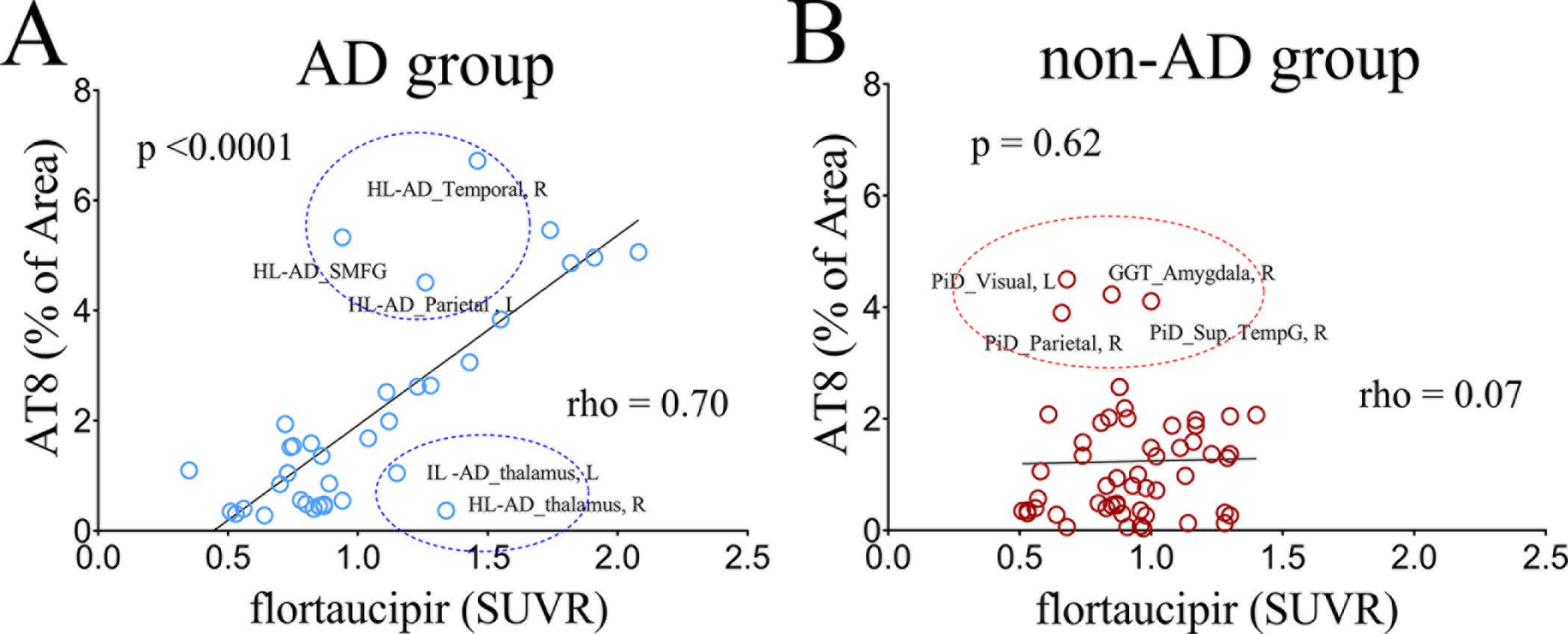
Correlations between neuropathological AT8 quantitation and Tau PET SUVRs. **A—**Comparison in the AD groups (non-AD control, low-AD, intermediate-AD, and high-AD. **B**—Comparison in the non-AD tauopathy group. Note a larger correlation in the AD groups. Some regions are not aligned with the main regression line (circled) implying a larger disparity or off-target regions, whereas others may imply more than the tau species involved in the high SUVR signal. Contrarily the non-AD group showed non-correlation. A distant point to the center line may be possible due to larger crosstalk between PHF1 and 3RT and 4RT tau species.

We observed a significant correlation between flortaucipir PET SUVR and quantitative AT8 burden in the ADNC group (rho = 0.70, p<0.0001) but no correlation between PET and AT8 burden in the non-AD tauopathies (rho = 0.07, p<0.62) **(Fig 6)**. However, there were some outlier regions for some patients in the ADNC group; the AD-high patient for example had relatively low flortaucipir uptake in some cortical regions with a high AT8 burden. On the contrary, we observed a higher-than-expected flortaucipir uptake in the thalamus given the low AT8 burden in the intermediate-AD and high-AD patients.

## Discussion

Our study demonstrates the complex relationship between flortaucipir uptake and histologically measured tau burden in AD-spectrum and non-AD spectrum tauopathies. As expected, there was an overall better correlation between flortaucipir PET uptake and histopathologically measured tau for the AD-spectrum patients than for the non-AD tauopathies. However, we did find regional discrepancies within the AD-spectrum patients, as well as good regional correspondence within some of the non-AD tauopathies.

From the study of individual patients, Tau-PET has been examined in the context of AD [42] and non-AD tauopathies [12], trying to link flortaucipir to neuropathological findings [9,43–46]. Overall, there is a lack of consistency between flortaucipir and neuropathology postmortem studies [12,47]. In our study, we found flortaucipir to have an overall good match with the patients with ADNC based on their Braak NFT stages. We found lower flortaucipir PET uptake in the low-AD and int-AD patients than in the AD-high patient. We also observed higher flortaucipir uptake and a larger percentage of AT8 tau deposition by area in the low-AD patient than in the Int-AD patient which may seem counter-intuitive. However, Braak NFT staging classifies tau deposition based on the distribution of PHF-tau, not on the burden of tau. Hence, although patients were classified as low-AD and Int-AD, regional tau burden, as measured with the AT8 antibody, was greater in the low-AD patient than the Int-AD patient. Another complicating factor that could explain this finding is that the low-AD patient also had argyrophilic grain disease (AGD), a 4R tauopathy that frequently co-deposits with other neurodegenerative proteins or diseases [48], with a prevalence reported as high as 31.3% in some centenarian series. In the low-AD patient, ADG was localized in the amygdala, hippocampus, and entorhinal cortex corresponding to a Saito stage II [49]. Hence, the increased tau detected by AT8 in the low-AD patient may be due to a combination of the higher burden of PHF tau plus the presence of AGD. We suspect that flortaucipir detected just the PHF tau but not the AGD in the low-AD patient, although the relative amount of detection of each pathological process is unclear.

There was significant, heterogeneous regional flortaucipir uptake across each non-AD tauopathy. This scattered uptake resulted in mismatched relationships between flortaucipir and tau burden in the non-AD tauopathies. For example, the patient with a clinical diagnosis of PSP-RS had higher uptake in the basal ganglia compared to the cortical regions in the patients with PSP-SL. The flortaucipir uptake in the PSP-RS patient more closely resembles uptake in the patient with GGT than the patient with PSP-SL, whose flortaucipir uptake was more like that of the patient with PiD. All five patients with non-AD tauopathy had higher flortaucipir uptake in the basal ganglia compared to cortical regions. Yet, the tau burden was not higher in the basal ganglia than in the cortical regions. This finding strongly supports non-tau off-target flortaucipir binding in the basal ganglia. It must be stressed however that this off-target binding is likely not specific to non-AD tauopathies and occur in patients with ADNC. Similar off-target binding in AD patients is likely being overshadowed by the relatively high amount of PHF tau binding by flortaucipir. Off-target binding of flortaucipir to MAO-A and B receptors, for example, would be expected to confound global uptake due to these receptors being present across the entire brain [50,51].

We also found evidence that flortaucipir detects at least some 4R tau although we cannot exclude the possibility that uptake is due to neurodegeneration-associated pathologies, as has been reported to occur with other tau PET markers such as 18F-THK5351 PET [52]. Regardless, we did find flortaucipir uptake to be higher in the frontal lobes of the PSP-SL patients compared to the PSP-RS patient which matched the higher burden in the frontal lobes of the PSP-SL patients observed with AT8. Given that all other factors are likely to be equally present in the frontal lobes of the PSP patients, it is not unreasonable to think that flortaucipir may be detecting some, albeit minimal, amounts of 4R tau.

In the other two non-AD tauopathies with PiD and GGT, we also observed some noteworthy results. AT8 in the PiD patient demonstrated a significantly higher tau burden in the temporal and parietal lobes compared to the other regions such as basal ganglia, thalamus, and midbrain. Yet flortaucipir uptake was relatively similar across all regions. This suggests that flortaucipir binding in Pick’s disease does not exactly mirror the burden of underlying 3R tau and may not detect all or even most, 3R tau. Globular glial tauopathy is a relatively newly described 4T tauopathy [53] with limited information on flortaucipir uptake reported in the literature [54]. As in PSP, we found a discrepancy between the tau burden measured with AT8 and flortaucipir uptake. AT8 displayed relatively high levels of tau in the amygdala compared to the other regions. This pattern of higher tau burden in this region was not mirrored with flortaucipir suggesting that the ligand is not detecting the 4R tau lesions in GGT.

This study has several possible limitations. First, the sample size of the cohort is relatively small. Second, the tau burden was measured with AT8, and although this is one of the most widely used antibodies in the world, it may not detect all molecular and tau isoform specie to the same degree. Third, pathological sampling, although robust, is still limited to a small area of brain tissue within a region, while flortaucipir regional SUVR data are generated from large ROIs. However, some noteworthy strengths of this study are the inclusions of different non-AD tauopathies (e.g., PSP, PiD, and GGT, as well as the two different clinical variants of PSP [PSP-RS and PSP-SL] associated with relatively different ratios of cortical to subcortical 4R tau. Also, we included all likelihoods of ADNC, low, intermediate, and high, and a patient designated as a control given the absence of neurodegeneration. Although arguably, it might have been better to include an elderly, cognitively normal patient (which we do not have), such patients are highly likely to have neurodegenerative changes, including ADNC, which would have limited our ability to compare the findings to our patients. The inclusion of the MELAS patient without any neurodegenerative changes is therefore a strength and not a weakness.

## Conclusions

With a bird’s eye view, quantitative neuropathological methods showed a good parallel with flortaucipir uptake in the ADNC group but a poor correlation between flortaucipir and non-AD tauopathies. However, a deeper dive into the relationship between flortaucipir PET and AD and non-AD tauopathies reveals a complex relationship between ligand uptake and underlying tau pathology.

## Abbreviations

AD: Alzheimer’s disease
ADNC: Alzheimer’s Disease Neuropathological Changes
MELAS: Mitochondrial Encephalopathy, Lactic Acidosis, and Stroke-like episodes
Int: intermediate Level
PSP: progressive supranuclear palsy
RS: Richardson syndrome
SL: speech/language variant
M: male
F: female. Ages are all reported in years. * *Median time of scan to death = 4 years*
